# An Inter-Subgenotypic PEDV Recombinant Harboring a Complete Structural Protein Cassette from the GIIb Lineage

**DOI:** 10.3390/v18101123

**Published:** 2026-10-10

**Authors:** Yue Chen, Lin Yuan, Wenqiang Wang, Dehu Sun, Lulu Chen, Jie Zhang, Zihan Guo, Pinnan Yan, Yana Chen, Junxia Lv, Yan Ma, Zhenbang Zhu, Wei Wen, Kegong Tian, Xiangdong Li

**Affiliations:** 1 Jiangsu Co-Innovation Center for Prevention and Control of Important Animal Infectious Diseases and Zoonoses, College of Veterinary Medicine, Yangzhou University, Yangzhou 225009, China; 19970371798@163.com (Y.C.); 008495@yzu.edu.cn (W.W.); 007583@yzu.edu.cn (Z.Z.); wenw@yzu.edu.cn (W.W.); 2 Beijing Sino-science Gene Technology Co., Ltd., Beijing 102629, China; yuan.lin@sslab.com.cn (L.Y.); sun.dehu@sslab.com.cn (D.S.); chen.lulu@sslab.com.cn (L.C.); zhang.jie1@sslab.com.cn (J.Z.); guo.zihan@sslab.com.cn (Z.G.); 3 Pudao (Beijing) Standard Technology Co., Ltd., Beijing 102629, China; yan.pinnan@sslab.com.cn (P.Y.); chen.yana@sslab.com.cn (Y.C.); lv.junxia@sslab.com.cn (J.L.); ma.yan@sslab.com.cn (Y.M.); 4 National Research Center for Veterinary Medicine, Luoyang 471000, China; 5 Jiangsu Key Laboratory of Zoonosis, Jiangsu Interdisciplinary Center for Zoonoses and Biosafety, Yangzhou University, Yangzhou 225009, China

**Keywords:** PEDV, spike protein, complete genome, phylogenetic analysis, recombination, molecular characterization

## Abstract

Porcine epidemic diarrhea virus (PEDV) poses a persistent threat to the global swine industry through the continual emergence of variants driven by mutation and recombination. Here, we report the isolation and genomic characterization of PEDV2−5, an inter-subgenotypic recombinant isolated from clinical samples collected in Jiangxi Province, China. Phylogenetic analysis based on both the complete genome and the full-length S gene placed PEDV2−5 within the GIIb subgenotypes, clustering closely with FL201 and GD-1. Recombination detection using seven independent algorithms in RDP5, corroborated by SimPlot and phylogenetic incongruence analysis, identified PEDV2−5 as a recombinant between a GIIa major parental-like (PEDV-LY) and a GIIb minor parental-like (GD-1), generating a 7931-nucleotide fragment (nt 20060–27990) that spans the complete structural protein cassette (S, ORF3, E, M, N) and the 3′-terminal portion of ORF1b. The recombinant has replication-competent Vero-E6 cells, reaching a peak titer of 5.03 log10 TCID_50_/mL at 36 h post-infection. Amino acid analysis revealed 98.49% identity between the PEDV2−5 S protein and the GIIb strain AJ1102, together with 13 unique substitutions and a “KKKG” insertion (residues 361–364), including two substitutions within neutralizing epitopes (A517F in COE and Y1374C in 2C10). Relative to AJ1102, the neutralizing epitopes were nearly completely conserved, whereas extensive divergence was observed against the classical GIa strain CV777. These findings highlight the importance of continued whole-genome surveillance for monitoring the emergence and spread of recombinant PEDV lineages and for informing future vaccine antigen-selection strategies.

## 1. Introduction

Porcine epidemic diarrhea virus (PEDV) is an enveloped, single-stranded, positive-sense RNA virus classified within the genus *Alphacoronavirus* of the family *Coronaviridae* [1,2]. With a genome of approximately 28 kb, the virus harbors seven open reading frames (ORFs) bounded by 5′ and 3′ UTRs. *ORF1a/b* direct the synthesis of replicase polyproteins; the other ORFs encode four structural proteins (*S*, *E*, *M*, *N*) and one accessory protein, ORF3 [3,4]. PEDV causes an acute, highly contagious enteric disease marked by watery diarrhea, vomiting, and dehydration, with neonatal mortality reaching 90–100%, leading to substantial economic losses worldwide [5,6,7]. In China, PEDV was first isolated in 1984, and infections were largely controlled through the administration of inactivated vaccines based on the classical CV777 strain until late 2010, when highly virulent variants emerged and caused extensive outbreaks with elevated mortality among suckling piglets [8,9].

The PEDV S glycoprotein is a homotrimeric type I membrane protein. It mediates viral attachment to and fusion with the host cell membrane, and constitutes the primary target of neutralizing antibodies [10,11,12]. Owing to its functional significance and high sequence variability, the S gene is widely employed as a molecular marker for assessing PEDV genetic diversity and evolutionary relationships [13,14,15]. Phylogenetic analyses based on the *S* gene have classified PEDV strains into two major genogroups: GI (classical) and GII (variant). The GI genogroup encompasses GIa and GIb subtypes, whereas the GII genogroup comprises GIIa, GIIb and GIIc subtypes. In 2014, new PEDV variants (OH851 and CH/HBQX/10) were identified in the USA and China. These strains, designated as S-INDEL, are hypothesized to have arisen from recombination events among classical and emerging PEDV strains [16]. Epidemiological surveillance conducted across multiple provinces in China has documented a dynamic shift in the predominant circulating subgenotypes: GIIa strains were dominant during the initial outbreaks of 2010–2013, followed by the emergence of GIIb variants, and most recent surveys indicate a progressive transition toward GIIc as the prevailing subtype [17]. This ongoing antigenic diversification has raised concerns regarding the protective efficacy of current commercial vaccines, which are based on the GIa (CV777) or GIIb (AJ1102) subtypes [18]. The S protein contains several well-defined neutralizing epitope regions: S10 (amino acids [aa] 19–220, excluding the signal peptide) within the N-terminal domain, the collagenase equivalent domain (COE, aa 499–638), SS2 (aa 748–755), SS6 (aa 764–771), and the C-terminal epitope 2C10 (aa 1368–1374). Amino acid substitutions and N-glycosylation changes in or proximal to these epitopes have been associated with altered antigenicity and immune evasion [14,17,18].

Recombination represents a major evolutionary mechanism driving the genetic diversification of coronaviruses, including PEDV [15,17,18]. The discontinuous nature of coronavirus RNA synthesis facilitates template-switching events during genome replication, and when two or more genetically distinct viral strains co-infect the same host cell, homologous recombination can generate chimeric progeny genomes [15]. Numerous inter-subgenotypic PEDV recombinant strains have been documented in China in recent years. For instance, the SXSL strain identified in mid-west China was shown to be a recombinant between GIIa and GIIb lineages, and its chimeric S protein conferred enhanced infectivity and pathogenicity compared with the parental CV777 backbone [15]. The GS2022 strain, belonging to the newly defined GIId subtype, was identified as a recombinant between JS-2-2015 (major parent) and CH-TP-E5-2018 (minor parent) and exhibited extensive antigenic variation in the S protein [8]. More recently, the GIIc recombinant strain HuB2025 was identified in Hubei Province, with recombination supported by seven independent algorithms in RDP5 [14]. It has also been proposed that the GIIc and GIId lineages themselves may have arisen through natural recombination events between S-INDEL strains and GIIa strains, highlighting the central role of recombination in shaping PEDV evolutionary trajectories [19]. Continuous genomic surveillance and characterization of emerging recombinant strains are therefore essential for monitoring PEDV evolutionary dynamics and informing vaccine development strategies.

In the present study, we report the isolation and comprehensive genomic characterization of a PEDV recombinant strain, designated PEDV2−5, obtained from Jiangxi, China. The objectives of this study were: (1) to determine the complete genome sequence of PEDV2−5 and perform phylogenetic analysis based on the full-length genome and S gene sequences, (2) to isolate the recombinant strain PEDV2−5 in vitro and characterize its biological properties, (3) to conduct detailed recombination analysis using multiple independent algorithms, (4) to assess amino acid variations in the S protein, particularly within known neutralizing epitope regions, and (5) to analyze the neutralization epitopes of the PEDV2−5 S protein in comparison with those of reference strains. These data contribute to the understanding of PEDV molecular evolution and may inform the development of improved diagnostic and vaccination strategies.

## 2. Materials and Methods

### 2.1. Clinical Sample Collection and Processing

The sample was collected from a pig farm in Jiangxi Province, China, in February 2026. And samples were collected from the intestines of 2-month-old piglets with diarrhea. We obtained informed consent from the farm owner for the use of the samples in this research. The intestinal tissues were homogenized. The homogenized intestinal tissue was then centrifuged at 4 °C at approximately 13,845× *g* to obtain the supernatant (Thermo, Sorvall Legend Micro 17R, Thermo Fisher Scientific, Waltham, MA, USA). The supernatant was filtered through a 0.22 μm membrane filter (Merck Millipore, Darmstadt, Germany) and stored at −80 °C until use.

### 2.2. Virus Isolation and Propagation

Vero-E6 cells (CCL-81, ATCC, Manassas, VA, USA) were maintained in Dulbecco’s Modified Eagle Medium (Gibco, Grand Island, NY, USA) supplemented with 10% fetal bovine serum (Gibco, 6126249) and 1% penicillin–streptomycin (Solarbio, Beijing, China, P1400) at 37 °C in a humidified atmosphere of 5% CO_2_. For virus isolation, Vero-E6 cell monolayers at approximately 90% confluence were washed three times with PBS and inoculated with 400 μL of filtered clinical sample supernatant. After adsorption at 37 °C for 2 h, the inoculum was removed, and cells were maintained in DMEM containing 0.3% tryptose phosphate broth (Sigma, Milwaukee, WI, USA, T8159), 5 μg/mL trypsin (Gibco, 25200-054), and 2% penicillin-streptomycin. The cells were monitored daily for the development of cytopathic effect (CPE). When approximately 80% CPE was observed after three blind passages, the culture was subjected to three freeze-thaw cycles, and the supernatant was collected for subsequent passages. The virus was continuously passaged until a stable CPE was observed.

### 2.3. RNA Extraction, RT-PCR, and Whole-Genome Sequencing

The virus was subjected to three successive rounds of plaque purification in Vero-E6 cells before RNA extraction and sequencing. Total RNA was extracted with TRIzol reagent (Tiangen, Beijing, China, DP424) and subjected to reverse transcription with HiScript III RT SuperMix for PCR (Vazyme, Nanjing, China, R323-01) with random hexamer primers. The primer sequences for detection are listed in Table 1. The PEDV-positive sample was subjected to whole-genome sequencing by Beijing Sino-science Gene Technology Co., Ltd. (Beijing, China), on an MGISEQ-2000 platform (MGI Tech, Shenzhen, China) with paired-end 150 bp (PE150) reads. Raw reads were quality-trimmed with fastp (v0.23.2). Clean reads were then mapped to the PEDV reference genome CV777 (GenBank AF353511.1) using BWA-MEM (v0.7.17), and a consensus sequence was generated with iVar (v1.3.1), requiring a minimum depth of 20× and a 75% majority-rule threshold at each nucleotide position. The final genome achieved 100% genome coverage with an average sequencing depth of 112,153×, with 100% of the genome covered at >20×. The 5′ and 3′ termini were confirmed by 5′/3′ RACE. The genome sequence of the PEDV2−5 was deposited in GenBank under accession number PZ939577.

### 2.4. Phylogenetic Analysis

Complete genome and S gene sequences of PEDV reference strains representing all major subgenotypes (GIa, GIb, S-INDEL, GIIa, GIIb, GIIc) were retrieved from the GenBank database. Detailed information on all reference strains is provided in Tables S1 and S2. Multiple sequence alignments were performed using the MAFFT algorithm (version 7.520) implemented in MEGA X software [20]. Phylogenetic trees were constructed using the maximum-likelihood (ML) method in IQ-TREE v3.1.2 with 1000 bootstrap replicates [21]. The best-fit nucleotide substitution model was determined using ModelFinder based on the Bayesian information criterion (BIC) values (GTR+F+R5 for 73 S gene ML tree, GTR+F+R5 for 46 complete genome ML tree) [22]. The resulting phylogenetic trees were visualized and annotated using iTOL v7 tool (https://itol.embl.de/) [23].

### 2.5. Recombination Analysis Methods

To detect potential recombination events in the PEDV2−5 genome, Recombination Detection Program version 5 (RDP5, v.5.67) was employed [24]. Seven independent algorithms were applied: RDP, GENECONV, BootScan, MaxChi, Chimaera, SiScan, and 3Seq. The analysis was performed using default parameters with a Bonferroni-corrected *p*-value threshold of 0.01. Only recombination events detected by three or more algorithms were considered credible. To further validate and localize the recombination events, similarity plot and BootScan analyses were conducted using SimPlot (version 3.5.1) [25], with PEDV2−5 as the query sequence and the identified parental strains as references. The sliding window was set to 200 bp with a step size of 20 bp. Additionally, phylogenetic trees were constructed for different genomic regions (non-recombinant and recombinant regions, as defined by the identified breakpoints) to verify phylogenetic incongruence. Trees were constructed using the ML method with the GTR+G+I model in IQ-TREE with 1000 bootstrap replicates.

### 2.6. S Protein Amino Acid Sequence Analysis

The deduced amino acid sequence of the PEDV2−5 S protein was aligned with that of representative PEDV reference strains, using ClustalW implemented in MEGA X. Amino acid substitutions, insertions, and deletions were identified by visual inspection and catalogued with reference to CV777 as the baseline. Particular attention was paid to substitutions within known neutralizing epitope regions: COE (aa 499–638), SS2 (aa 748–755), SS6 (aa 764–771), 2C10 (aa 1368–1374), and S10 (aa 19–220) [26].

### 2.7. Indirect Immunofluorescence Assay

Vero-E6 cells grown on coverslips were infected with PEDV2−5 and fixed with 4% paraformaldehyde at various time points post-infection (9 hpi, 12 hpi). Cells were permeabilized with 0.5% Triton X-100 and incubated with a mouse anti-PEDV S protein monoclonal antibody (1:1000 dilution) (a generous gift from Prof. Yongchang Cao from Sun Yat-sen University) [27], followed by FITC-conjugated goat anti-mouse IgG secondary antibody (1:1000 diluted) (Thermo, A-11029). Nuclei were counterstained with DAPI (1:2000) (Beyotime, Shanghai, China, NG0131). Images were captured using a fluorescence microscope.

### 2.8. Viral Growth Kinetics

Vero-E6 cells seeded in 6-well plates were infected with PEDV2−5 at a multiplicity of infection (MOI) of 0.1. Cell supernatants were collected at 0, 6, 12, 18, 24, 30, 36, 42 and 48 h. Virus titers were determined by the 50% tissue culture infectious dose (TCID_50_) assay on Vero-E6 cells and calculated using the Reed-Muench method [28]. All experiments were performed in triplicate, and data were expressed as mean ± standard deviation (SD).

### 2.9. Statistical Analysis

The results are expressed as mean ± SD values.

## 3. Results

### 3.1. Complete Genome Characterization and Phylogenetic Analysis

**Figure 1:**
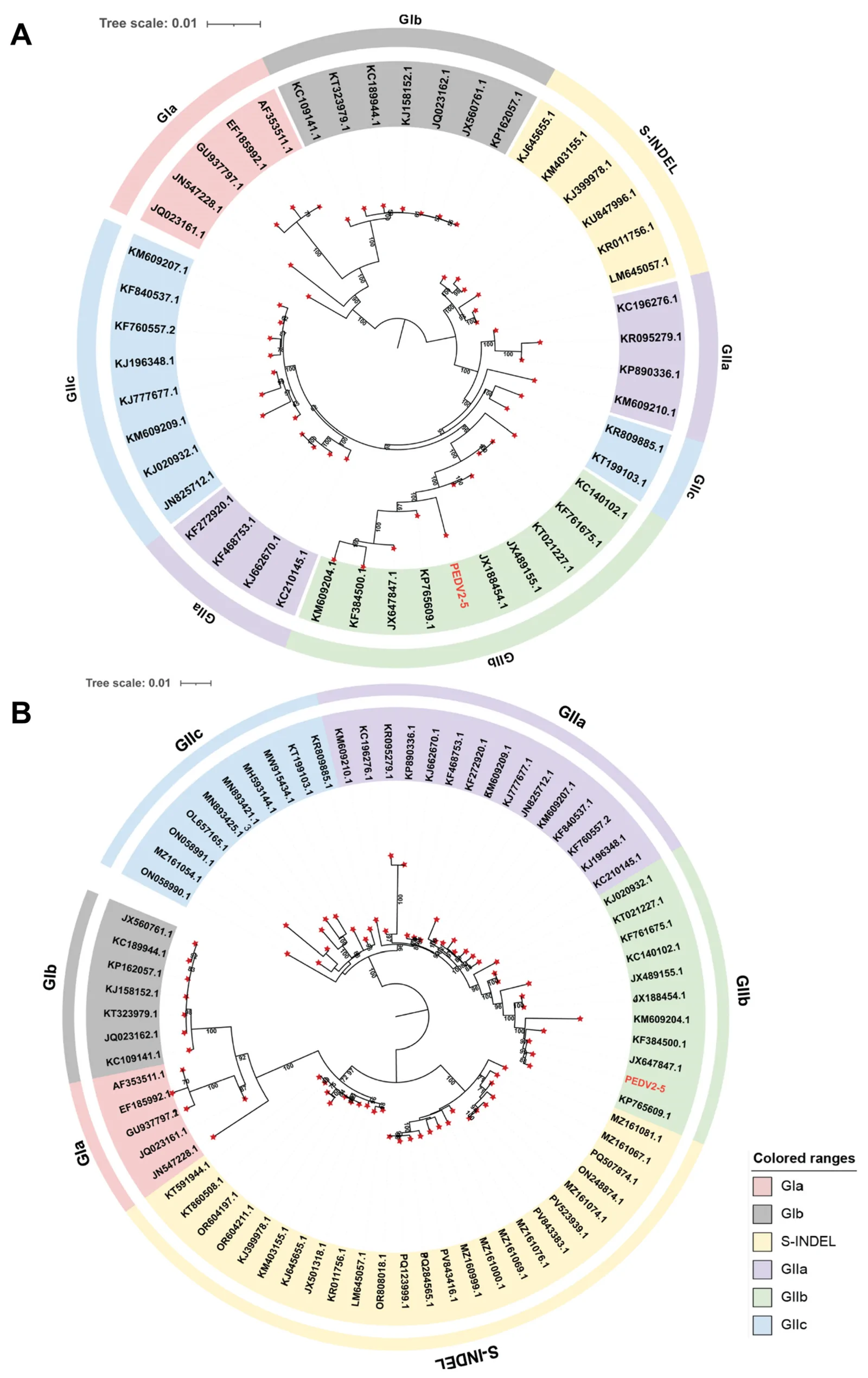
Phylogenetic analysis of PEDV2−5 based on complete genome and full-length S gene sequences. (**A**) Maximum-likelihood (ML) phylogenetic tree constructed from complete genome sequences of PEDV2−5 and 45 representative PEDV reference strains belonging to subgenotypes GIa, GIb, S-INDEL, GIIa, GIIb, and GIIc. (**B**) ML phylogenetic tree constructed from full-length S gene sequences and 72 representative PEDV reference strains belonging to subgenotypes GIa, GIb, S-INDEL, GIIa, GIIb, and GIIc. PEDV2−5 is highlighted in red (red text and red star). Scale bars indicate nucleotide substitutions per site. The trees were constructed using IQ-TREE with the best-fit substitution model determined by ModelFinder, and were visualized using iTOL.

To elucidate the phylogenetic relationship of PEDV2−5 with representative PEDV strains, maximum-likelihood (ML) phylogenetic trees were constructed based on the complete genome sequences and the full-length S gene sequences. In the complete genome phylogenetic tree (Figure 1A, Table S1), the reference strains were resolved into distinct clades corresponding to the established subgenotypes: GIa, GIb, S-INDEL, GIIa, GIIb, and GIIc. PEDV2−5 belongs to the GIIb subtype and clusters closely with FL201 (KP765609.1). In the full-length S gene phylogenetic tree (Figure 1B, Table S2), PEDV2−5 was also placed within the GIIb clade, clustering closely with FL201 (KP765609.1) and GD-1 (JX647847.1).

### 3.2. Biological Characterization of PEDV2−5

**Figure 2:**
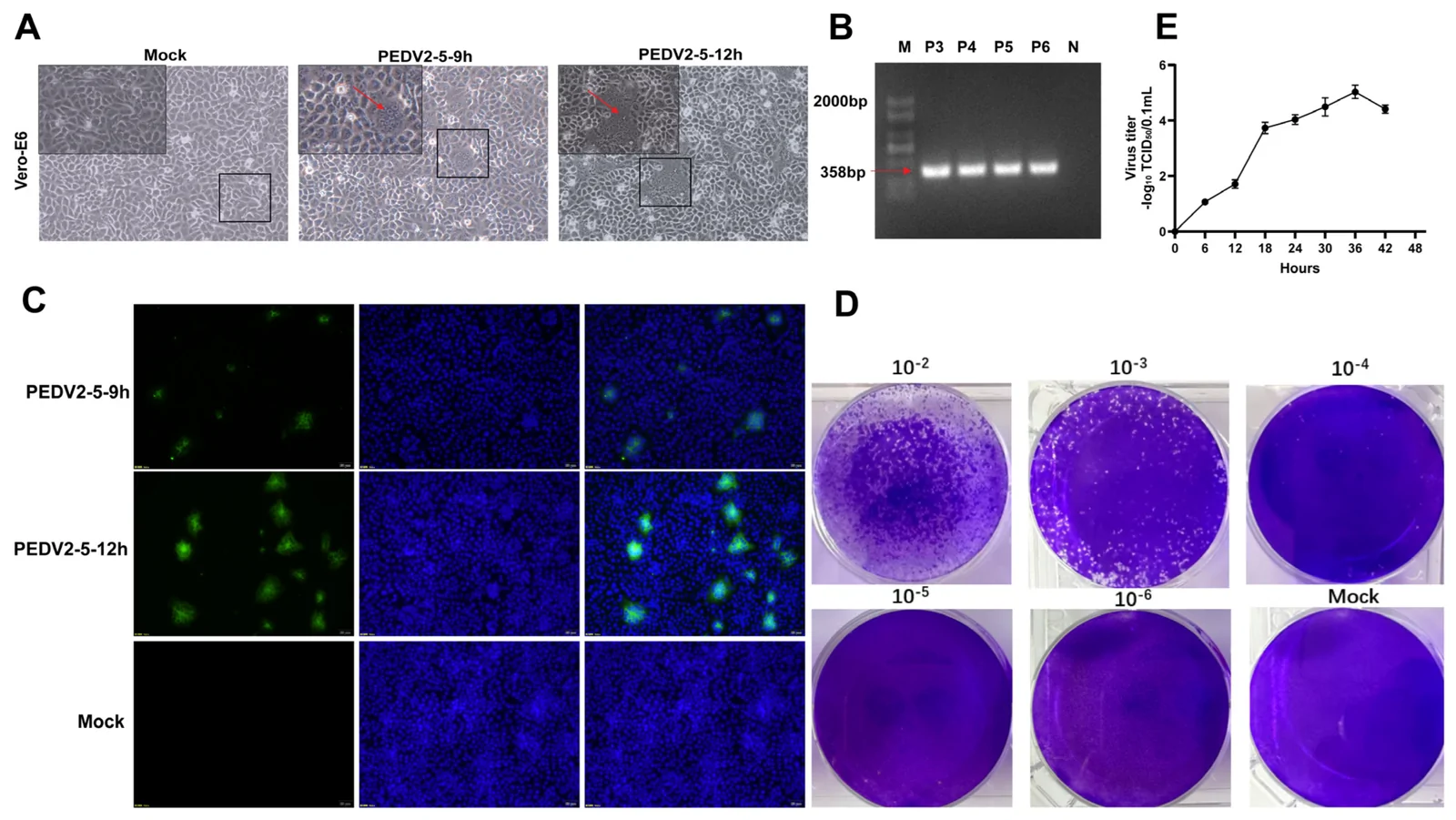
Biological characterization of PEDV2−5. (**A**) Cytopathic effect (CPE) in Vero-E6 cells infected with PEDV2−5 at 9 hpi. Mock-infected cells are shown as a control. Scale bar = 100 μm. Red arrows indicate lesions caused by PEDV2−5 infection. (**B**) Detection of PEDV2−5 N gene using RT-PCR. Red arrow indicates the target band. (**C**) Indirect immunofluorescent assay (IFA) of PEDV2−5-infected Vero-E6 cells using a mouse anti-PEDV S protein monoclonal antibody (green). Nuclei were counterstained with DAPI (blue). Scale bar = 100 μm. (**D**) A plaque assay for PEDV2−5-infected Vero-E6 cells. (**E**) One-step growth curve of PEDV2−5 in Vero-E6 cells at an MOI of 0.1. Virus titers were determined by TCID_50_ assay at the indicated time points. Data are presented as mean ± SD from three independent experiments.

PEDV2−5 induced typical CPE, characterized by cell rounding, fusion, syncytium formation, and eventual detachment from the monolayer, starting at approximately 9 hpi (Figure 2A). In contrast, mock-infected Vero-E6 cells maintained a normal monolayer morphology throughout the observation period. RT-PCR amplification of a partial fragment of the PEDV N gene confirmed the presence of viral RNA in infected cultures (Figure 2B) (primers are listed in Table 1). IFA using a monoclonal antibody against the PEDV S protein revealed strong, specific cytoplasmic fluorescence in PEDV2−5-infected Vero-E6 cells at 9 hpi and 12 hpi, confirming viral protein expression (Figure 2C). No fluorescence signal was detected in mock-infected control cells. Plaque assay revealed visible plaques at dilutions ranging from 10^−1^ to 10^−5^, whereas no plaques were observed in the mock-infected control group (Figure 2D). The one-step growth curve of PEDV2−5 in Vero-E6 cells showed that the viral titer increased exponentially during 12–18 hpi, reaching a peak titer of 5.03 log_10_ TCID_50_/mL at 36 hpi, after which the titer plateaued and gradually declined (Figure 2E).

### 3.3. Recombination Analysis

**Figure 3:**
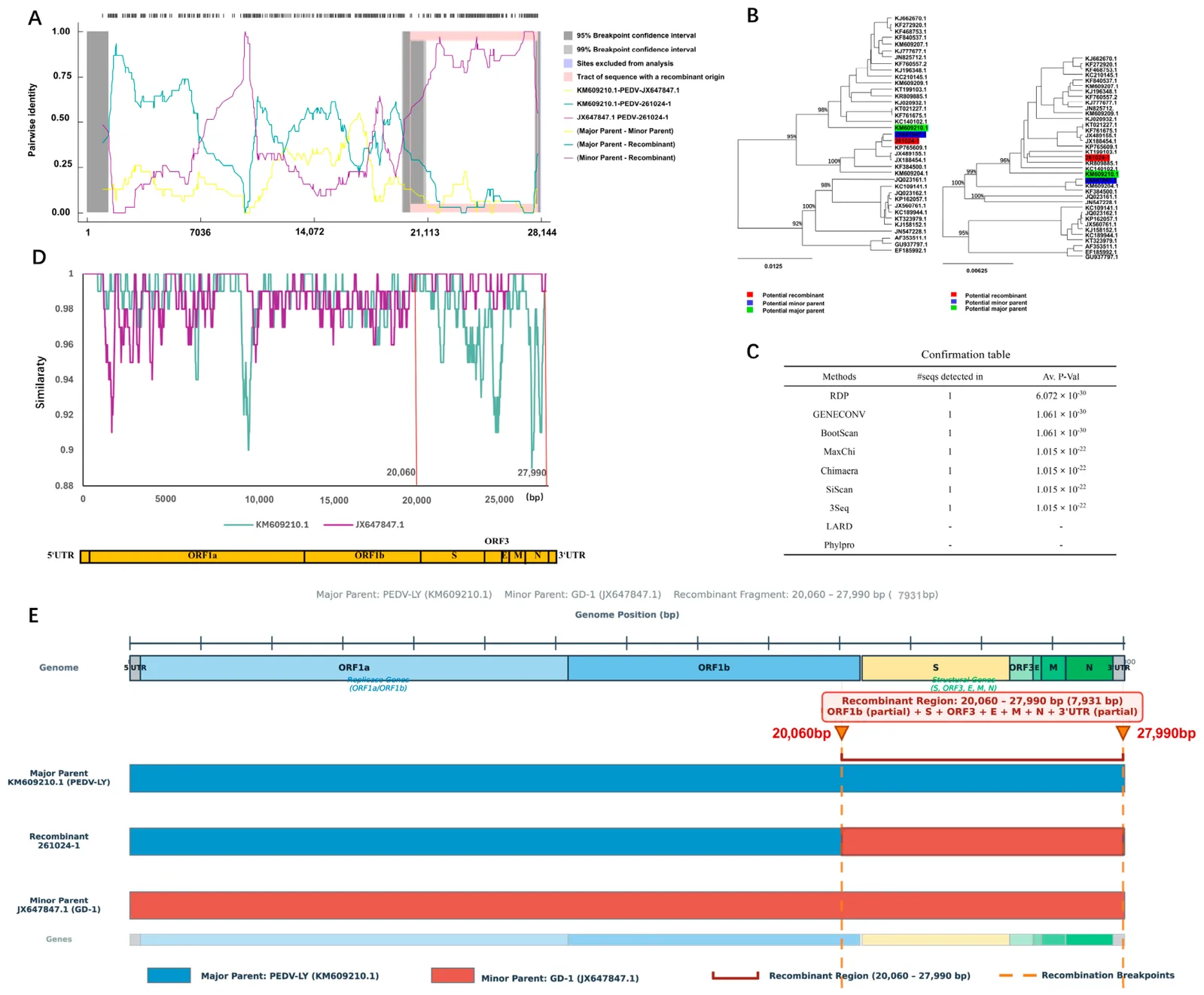
Recombination analysis of PEDV2−5. (**A**) Schematic representation of the recombination event identified by RDP5. The major parental-like PEDV-LY (KM609210.1, yellow), the recombinant PEDV2−5, and the minor parental-like GD-1 (JX647847.1, purple) strains are shown. The recombination region is indicated by the red bracket. (**B**) Phylogenetic trees constructed from the non-recombinant region (nt 1–20059, left) and the recombinant region (nt 20060–27990, right). PEDV2−5 is indicated in red, PEDV-LY in green, and GD-1 in blue. Bootstrap values (≥70%) are shown at nodes. (**C**) Statistical support for the recombination event from seven independent algorithms in RDP5. The dashed red line indicates the Bonferroni-corrected significance threshold (*p* = 0.01). (**D**) SimPlot similarity and BootScan analyses of PEDV2−5 with PEDV-LY (green) and GD-1 (purple) as putative parental strains. The Y-axis represents pairwise identity (upper panel) or bootstrap support (lower panel). The X-axis represents nucleotide position along the PEDV2−5 genome. The recombination breakpoints at nt 20060 and 27990 are indicated by red vertical dashed lines. The horizontal bars above the curves illustrate the genomic organization of PEDV2−5, with the recombinant region highlighted in red. Sliding window: 200 bp; step size: 20 bp. (**E**) Recombination pattern of PEDV2−5.

To assess whether the isolated strain PEDV2−5 was a recombination strain, comprehensive recombination analysis was performed using RDP5 and SimPlot. RDP5 analysis identified PEDV2−5 as a statistically significant recombinant, with PEDV-LY (KM609210.1) PEDV2−5 identified as the major parental lineage and GD-1 (JX647847.1) as the minor parental lineage (Figure 3A). To further validate the recombination event, phylogenetic trees were independently constructed for the non-recombinant region (nt 1–20059) and the recombinant region (nt 20060–27990). In the non-recombinant region tree, PEDV2−5 clustered tightly with the major parental-like PEDV-LY and other GIIa strains, confirming the GIIa backbone origin. In striking contrast, in the recombinant region tree, PEDV2−5 shifted its phylogenetic position to cluster with the minor parental-like GD-1 and other GIIb strains. This clear phylogenetic incongruence strongly corroborated the RDP5 and SimPlot results, confirming that PEDV2−5 is an inter-subgenotypic recombinant between GIIa (backbone) and GIIb (structural protein cassette) lineages (Figure 3B). Crucially, this recombination event was supported by seven independent detection algorithms, yielding extremely low *p*-values: RDP (*p* = 6.072 × 10^−30^), GENECONV (*p* = 1.061 × 10^−30^), BootScan (*p* = 1.061 × 10^−30^), MaxChi (*p* = 1.015 × 10^−22^), Chimaera (*p* = 1.015 × 10^−22^), SiScan (*p* = 1.015 × 10^−22^), and 3Seq (*p* = 1.015 × 10^−22^) (Figure 3C, Table S3).

SimPlot analysis using PEDV2−5 as the query sequence, with PEDV-LY and GD-1 as the putative parental strains, was performed to precisely map the recombination breakpoints (Figure 3D). The similarity plot and BootScan analysis concordantly identified the recombination breakpoints at nucleotide positions 20060 and 27990, defining a recombination fragment of 7931 bp. Within this recombination region (nt 20060–27990), PEDV2−5 exhibited substantially higher sequence similarity to the minor parental-like GD-1 (GIIb) than to the major parental-like PEDV-LY (GIIa), while outside this region, the similarity to PEDV-LY was markedly higher, consistent with the RDP5 findings. Furthermore, we confirmed these recombinant breakpoints providing read-level mapping and using RT-PCR and Sanger sequencing. The results revealed the exact breakpoint (Figures S1 and S2, Table S4). Integrating the genomic recombination profiles, we mapped the recombination pattern of PEDV2−5. The resulting schematic clearly demonstrates that PEDV2−5 is a recombinant virus featuring a partial substitution in ORF1b and complete replacement of the structural genes (S, E, M, N) (Figure 3E).

### 3.4. S Protein Amino Acid Variation Analysis

**Figure 4:**
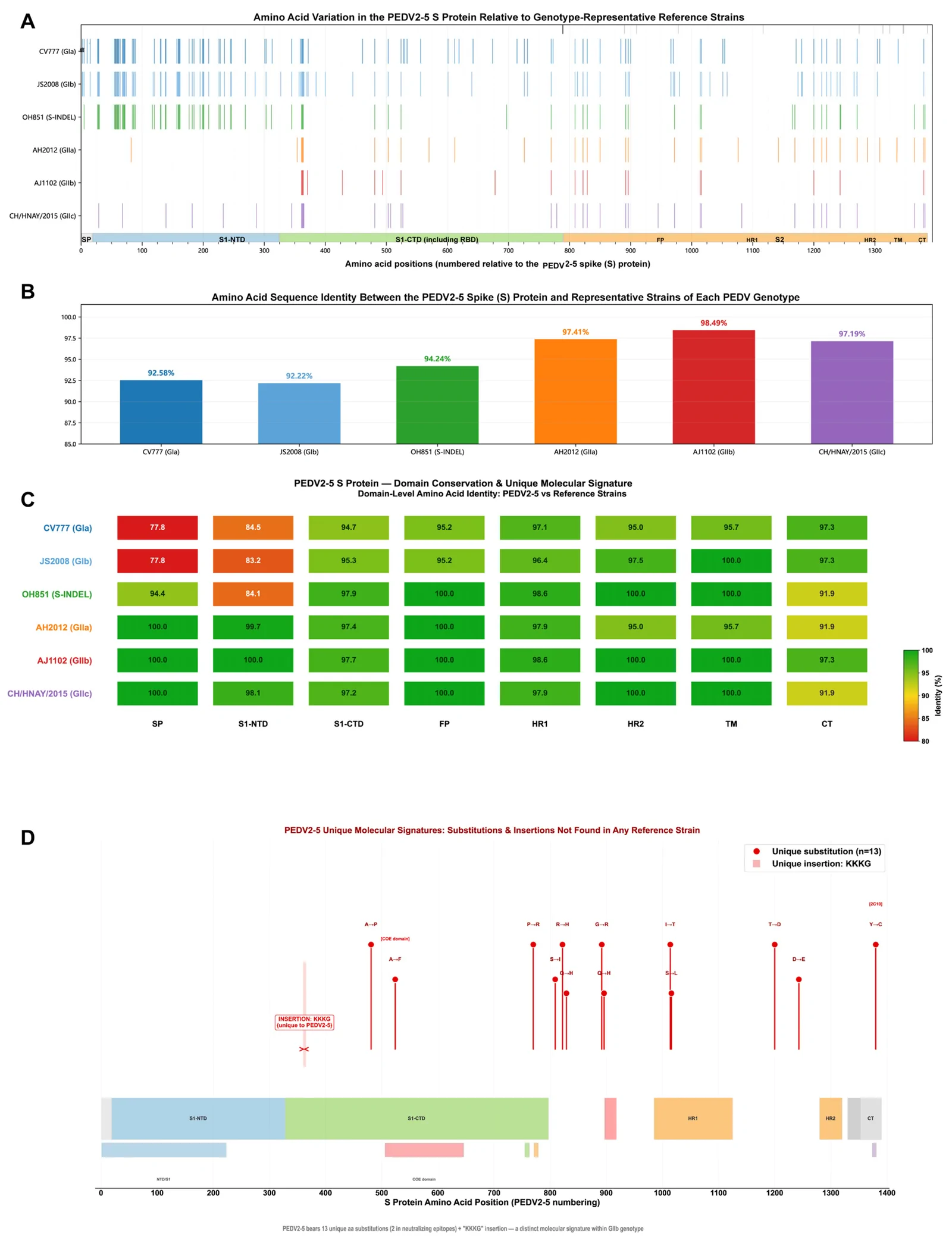
Amino acid variation analysis of PEDV S protein. (**A**) Amino acid variation in the PEDV2−5 S protein relative to reference strains. (**B**) Amino acid sequence identity between the PEDV2−5 S protein and reference strains. (**C**) Conservation comparison of S protein in different domains between PEDV2−5 and reference strains. (**D**) Unique molecular signature of S protein between PEDV2−5 and reference strains.

Given the critical role of the S protein as the major antigenic determinant of PEDV, the deduced amino acid sequence of the PEDV2−5 S protein was aligned and compared with representative reference strains from all major subgenotypes, including CV777 (GIa), JS2008 (GIb), OH851 (S-INDEL), AH2012 (GIIa), AJ1102 (GIIb), and CH/HNAY/2015 (GIIc) (Table 2). The results showed that the S protein of PEDV2−5 exhibited the highest homology with AJ1102, with a minimal overall difference of 98.49%. In contrast, greater divergence was observed compared to JS2008 (GIb), CV777 (GIa), and OH851 (S-INDEL), with homology ranging from 92.22% to 94.24%. The amino acid variations between the S protein of PEDV2−5 and those of various subgenotypes were primarily concentrated in the S1-NTD region (Figure 4A,B). Notably, PEDV2−5 and AJ1102 achieved 100% amino acid identity across five domains—SP, NTD, FP, HR2, and TM—representing the highest match among the six strains analyzed. Intriguingly, the homology of the PEDV2−5 S protein domains with the S-INDEL strain (OH851) fell between GI and GII genotypes: SP = 94.4%, NTD = 84.1% (closer to GI), whereas CTD = 97.9% and FP/HR2/TM = 100% (closer to GII). This pattern may suggest a “chimeric” intermediate profile for S-INDEL (Figure 4C).

Subsequently, we analyzed specific amino acid substitutions in the PEDV2−5 S protein. Compared with the six reference strains, PEDV2−5 harbored 13 unique amino acid substitutions and a distinct “KKKG” insertion (residues 361–364 within the S1-NTD region). Crucially, two of these variations occurred within neutralizing epitopes: a substitution at position 517 in the COE domain (Phe) and a substitution at position 1374 within the 2C10 epitope (Cys) (Figure 4D).

### 3.5. Neutralizing Epitope Comparison of the PEDV2−5 S Protein

**Figure 5:**
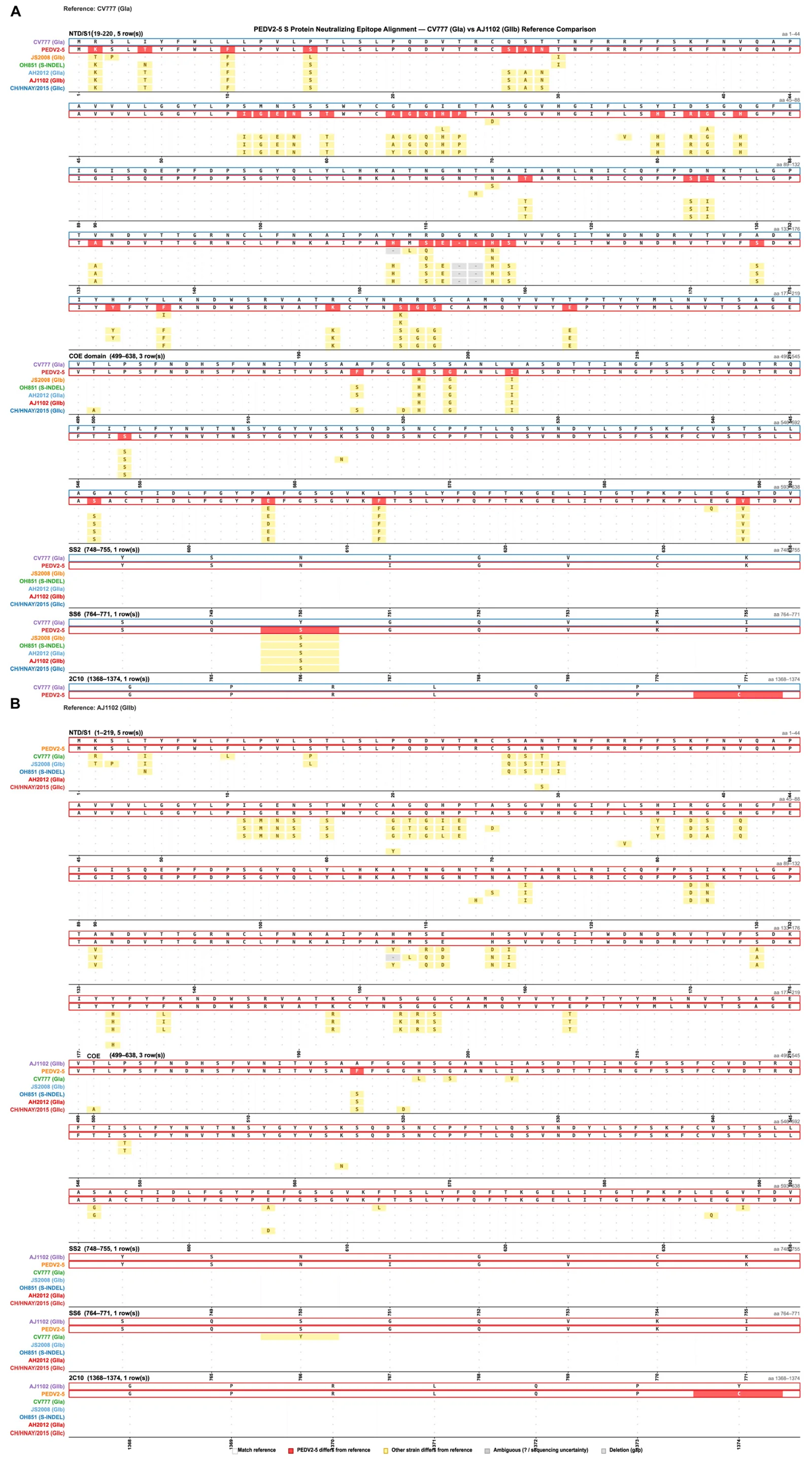
Neutralizing epitope alignment of the PEDV2−5 S protein. (**A**) CV777 (GIa) as reference. (**B**) AJ1102 (GIIb) as reference. Five known neutralizing epitopes (NTD/S1, COE, SS2, SS6, and 2C10) were aligned across PEDV2−5 and six reference strains. Large epitopes are displayed in wrapped segments. Non-reference/non-PEDV2−5 residues identical to the reference are shown as dots. Red: PEDV2−5 differs from reference. Yellow: other strains differ from reference. White: identical to reference. Gray: deletion or ambiguous. PEDV2−5 row is highlighted with a red border.

Amino acid sequences of five known neutralizing epitopes, NTD/S1 (aa 1–219), COE (aa 499–638), SS2 (aa 748–755), SS6 (aa 764–771), and 2C10 (aa 1368–1374), which were aligned across PEDV2−5, and six reference strains representing subgenotypes GIa, GIb, S-INDEL, GIIa, GIIb, and GIIc, using CV777 (GIa) and AJ1102 (GIIb) as alternative reference sequences. When aligned against the classical reference strain CV777 (GIa), PEDV2−5 exhibited extensive divergence across the neutralizing epitopes (Figure 5A). Within the NTD/S1 epitope (aa 19–220), PEDV2−5 differed from CV777 at 40 amino acid positions, the highest number among all five epitopes. These substitutions were distributed throughout the epitope, with a notably dense cluster spanning positions 55–68 (S55I, M56G, N57E, S58N, S60T, G64A, T65G, G66Q, I67H, E68P), and included a two-residue deletion at positions 156–157 (G156-K157) adjacent to a D158H substitution. Additional substitutions clustered at positions 152–159 (Y152H, R154S, D155E, G156-K157 deletion, D158H, I159S) and positions 196–198 (R196S, R197G, S198G), with scattered substitutions throughout the remaining region (R2K, I5T, L10F, P15S, Q27S, S28A, T29N, Y80H, D82R, S83G, Q85H, I116T, D126S, N127I, V134A, A174S, H179Y, L182F, R192K, T206E). In the COE domain (aa 499–638), the primary target of neutralizing antibodies, nine substitutions were identified relative to CV777: A517F, L521H, S523G, V527I, T549S, G594S, A605E, L612F, and I635V. Among the short linear epitopes, SS2 (aa 748–755) was fully conserved across all seven strains, whereas SS6 (aa 764–771) harbored a Y766S substitution and 2C10 (aa 1368–1374) carried a Y1374C substitution.

When aligned against the GIIb reference strain AJ1102, PEDV2−5 exhibited near-complete conservation across all five neutralizing epitopes, consistent with their shared GIIb subgenotypes (Figure 5B). Specifically, the NTD/S1 epitope (aa 1–219), SS2 (aa 748–755), and SS6 (aa 764–771) were fully identical between PEDV2−5 and AJ1102. Only two amino acid substitutions were identified across the five epitopes: A517F within the COE domain (aa 499–638) and Y1374C within the 2C10 epitope (aa 1368–1374). Notably, the Y1374C substitution was exclusive to PEDV2−5 among all seven strains examined. Collectively, these results indicate that the 51 residue-level differences observed between PEDV2−5 and CV777 predominantly reflect GIIb-lineage polymorphisms rather than PEDV2−5-specific alterations, as PEDV2−5 retains the core GIIb neutralizing epitope architecture with only two substitutions relative to the prototypical GIIb strain AJ1102.

## 4. Discussion

In this study, we report the isolation and comprehensive genomic characterization of PEDV2−5, an inter-subgenotypic PEDV recombinant strain originating from a recombination event between a GIIa major parental-like (PEDV-LY, KM609210.1) and a GIIb minor parental-like (GD-1, JX647847.1). The recombination event, robustly supported by seven independent algorithms implemented in RDP5 with extremely low *p*-values, generated a 7931-nucleotide recombinant fragment (nt 20060–27990) that spans the complete coding sequences of all five structural protein genes (S, ORF3, E, M, N), together with the 3′-terminal portion of ORF1b. Phylogenetic incongruence between the non-recombinant and recombinant genomic regions independently validated the recombination hypothesis, with PEDV2−5 clustering within the GIIa clade in the backbone region while shifting to the GIIb clade in the structural gene region. This recombination event involves a large-scale exchange of structural protein-coding sequences between two distinct GII subgenotypes: the structural protein genes are inherited as a contiguous block from the minor parental GIIb lineage, whereas the replicase backbone originates from the major parental GIIa lineage. To our knowledge, this pattern expands the growing repertoire of PEDV recombinant variants documented in China [14,15,16].

The recombination event characterized in PEDV2−5 differs from previously documented PEDV recombinants in several important respects. First, while many reported recombinants, such as SXSL (GIIa × GIIb) and HuB2025 (GIIc), harbor breakpoints confined to or flanking the S gene alone, PEDV2−5 acquired the complete structural gene cassette (S-ORF3-E-M-N) from GD-1 in what appears to be a single, large-scale template-switching event [14,17]. Second, the recombination fragment in PEDV2−5 (7931 bp) is substantially larger than the fragments typically reported for PEDV recombinants. For comparison, the GS2022 (GIId) recombinant involved a more restricted genomic segment centered on the S gene [18], and the recently described HuB2025 strain similarly carried a partial rather than complete structural gene replacement [16]. Third, the location of the recombination breakpoints, at the ORF1b/S gene junction (nt 20060) and the N/3′UTR boundary (nt 27990), is noteworthy, as these intergenic regions correspond to recognized recombination hotspots in coronavirus genomes. The presence of transcription regulatory sequences (TRS) and stable RNA secondary structures at these junctions likely facilitates polymerase pausing and template switching, thereby promoting homologous recombination between co-infecting viral genomes [17]. The co-circulation of GIIa and GIIb strains in Chinese swine herds, as documented by recent epidemiological surveys, provides the necessary ecological context for such co-infection and recombination events to occur.

Biological characterization of PEDV2−5 demonstrated that the recombinant virus retains robust replication capacity in vitro. The virus was successfully isolated from clinical samples and propagated in Vero-E6 cells, producing typical CPE, including cell rounding, fusion, syncytium formation, and monolayer detachment, by 9 h post-infection. The one-step growth curve revealed exponential replication between 12 and 18 hpi, with a peak titer of 5.03 log_10_ TCID_50_/mL at 36 hpi, after which the titer plateaued and gradually declined. This growth kinetic profile is broadly consistent with those reported for other GIIb field strains, suggesting that the GIIa-derived replicase backbone and the GIIb-derived structural proteins are functionally compatible and do not impose a significant fitness cost on viral replication in cell culture. IFA using an anti-PEDV S protein monoclonal antibody confirmed expression of the GIIb-lineage S protein, and plaque assay results demonstrated that PEDV2−5 forms visible plaques at dilutions up to 10^−5^, further supporting its replicative competence. However, no comparator strain was available for parallel growth-curve analysis (the parental strains PEDV-LY and GD-1 were not available), so no conclusion about enhanced or altered replication efficiency is drawn. And whether the chimeric nature of the PEDV2−5 genome, combining a GIIa replicase with GIIb structural proteins, confers any selective advantage or disadvantage relative to the parental strains in vivo remains to be determined.

Given that the S protein is the principal determinant of PEDV antigenicity and the primary target of host neutralizing antibodies, the complete replacement of the S gene from a GIIa to a GIIb lineage in PEDV2−5 may carry immunological implications. Amino acid alignment of the PEDV2−5 S protein with representative strains from all major subgenotypes revealed the highest overall homology with the GIIb vaccine strain AJ1102 (98.49% identity), consistent with the GIIb origin of the recombinant S gene. Detailed analysis of five well-characterized neutralizing epitopes, NTD/S1, COE, SS2, SS6, and 2C10, showed that PEDV2−5 exhibits near-complete conservation with AJ1102, with only two amino acid substitutions identified across all five epitopes: A517F within the COE domain and Y1374C within the 2C10 epitope. Of particular note, the Y1374C substitution was unique to PEDV2−5 among all seven strains examined and resides within a C-terminal epitope recognized by neutralizing monoclonal antibodies [19]. In contrast, alignment against the classical CV777 (GIa) reference strain revealed these amino acid differences (51 substitutions and a two-residue deletion at positions 156–157) distributed across all five epitope regions, with the highest divergence concentrated in the NTD/S1 domain (40 substitutions). Additionally, PEDV2−5 harbors a unique ‘KKKG’ insertion at residues 361–364 within the S1-NTD region, a feature not observed in any of the six reference strains examined. While the functional significance of this insertion remains unclear, its location within the hypervariable NTD region, which has been implicated in sialic acid binding and host tropism determination in other coronaviruses, warrants further investigation.

Several limitations of the present study should be acknowledged. First, the pathogenicity and virulence of PEDV2−5 were not evaluated in vivo using a neonatal piglet challenge model. Therefore, the clinical significance of this recombinant strain, including its capacity to cause diarrhea, intestinal atrophy, and mortality relative to contemporary field strains, remains to be determined. Second, virus neutralization assays using convalescent or hyperimmune sera raised against current commercial vaccine strains (CV777-based GIa and AJ1102-based GIIb vaccines) were not performed, which precludes direct assessment of whether the identified S protein amino acid substitutions compromise vaccine-elicited protective immunity. Third, this study characterized a single recombinant isolate from one geographical location (Jiangxi Province); broader surveillance incorporating temporally and geographically diverse clinical samples would be necessary to assess the prevalence, transmission dynamics, and epidemiological significance of this recombination pattern. Fourth, the parental-like strains PEDV-LY and GD-1 were not available for parallel phenotypic comparison, limiting our ability to attribute specific biological properties to the recombination event per se versus lineage-specific traits.

The emergence of PEDV2−5, together with other recently documented recombinant variants such as SXSL, GS2022, and HuB2025, underscores the ongoing and dynamic nature of PEDV evolution in Chinese swine herds. The identification of a recombination pattern involving the wholesale exchange of the complete structural protein cassette between co-circulating lineages raises several considerations for PEDV control strategies. First, molecular diagnostic assays targeting the S gene alone may not adequately capture the full genomic complexity of recombinant strains, highlighting the value of whole-genome sequencing as a complement to S gene-based genotyping in surveillance programs. Second, the progressive antigenic divergence of circulating strains from historical vaccine seed viruses, driven by both mutation accumulation and recombination-mediated epitope replacement, necessitates periodic re-evaluation of vaccine strain selection and may ultimately warrant the development of multivalent or lineage-matched vaccines. Third, the demonstration that PEDV2−5 emerged through recombination between GIIa and GIIb lineages co-circulating in the same geographical region reinforces the importance of biosecurity measures aimed at preventing the introduction of genetically disparate strains into naive herds, where co-infection creates conditions permissive for recombination. Continued genomic surveillance integrating whole-genome sequencing with systematic phenotypic characterization will be essential to monitor the evolutionary trajectory of PEDV, identify emerging antigenic variants, and inform evidence-based vaccine development strategies.

## 5. Conclusions

In conclusion, we successfully isolated an emerging inter-subgenotypic PEDV recombinant strain (PEDV2−5) and characterized its complete genome. The recombinant virus exhibited replication-competent in Vero-E6 cells. Amino acid analysis revealed near-complete conservation of GIIb-lineage neutralizing epitopes, with only two substitutions (A517F in COE and Y1374C in 2C10) relative to the prototype GIIb strain AJ1102, but marked divergence from the classical GIa vaccine strain CV777. This recombinant strain expands the known repertoire of PEDV recombination patterns and provides a valuable model for studying how single template-switching events generate novel genomic configurations, supporting the need for continuous whole-genome surveillance.

## Figures and Tables

**Table 1:** Primers for the detection of PEDV.

Primer Name	Primer Sequences (5′–3′)
PEDV-N-F	AACCTGGCCATTTCAATAAGGAGTT
PEDV-N-R	ATAGTGGGCAGGATGTTACGCTTAG

**Table 2:** Reference strains of different subgenotypes of PEDV.

Subtype	Strain Name	GenBank No.
GIa	CV777	AF353511.1
GIb	JS2008	KC109141.1
S-INDEL	OH851	KJ399978.1
GIIa	AH2012	KC210145.1
GIIb	AJ1102	JX188454.1
GIIc	CH/HNAY/2015	KR809885.1

## Data Availability

The data that supports the findings of this study are available in the Supplementary Materials of this article.

## Supplementary Materials

The following supporting information can be downloaded at: https://www.mdpi.com/article/10.3390/v18101123/s1, Figure S1: Read-level mapping across the recombination breakpoints of PEDV2−5 visualized by Integrative Genomics Viewer (IGV); Figure S2: RT-PCR and Sanger confirmation for recombination breakpoint of PEDV2−5; Table S1. Reference strains for constructing the phylogenetic tree based on whole-genome; Table S2: Reference strains for constructing the phylogenetic tree based on the S gene; Table S3 Information of recombination events detected by using RDP5; Table S4 Primer information of detecting the breakpoint of PEDV2−5.
