# Supplementary material for: An Inter-Subgenotypic PEDV Recombinant Harboring a Complete Structural Protein Cassette from the GIIb Lineage
Source: Viruses. 2026 Oct 10;18(10):1123. doi: 10.3390/v18101123 (PMC13709889; doi:10.3390/v18101123)
Supplement: Supplementary file 1 [file viruses-18-01123-s001.zip › Supplementary Figure.pdf]

Supplementary Figure

A

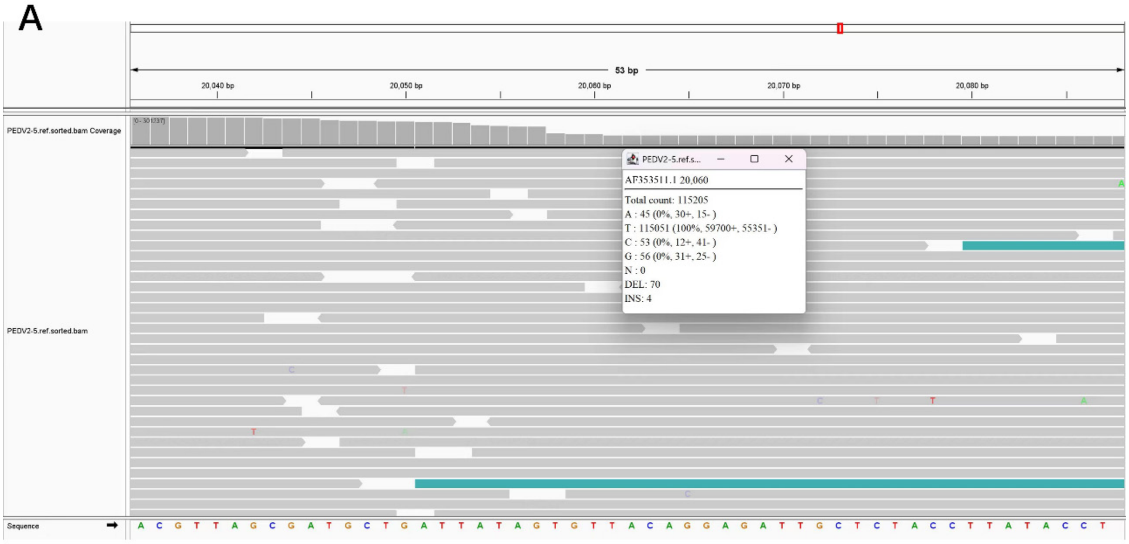

B

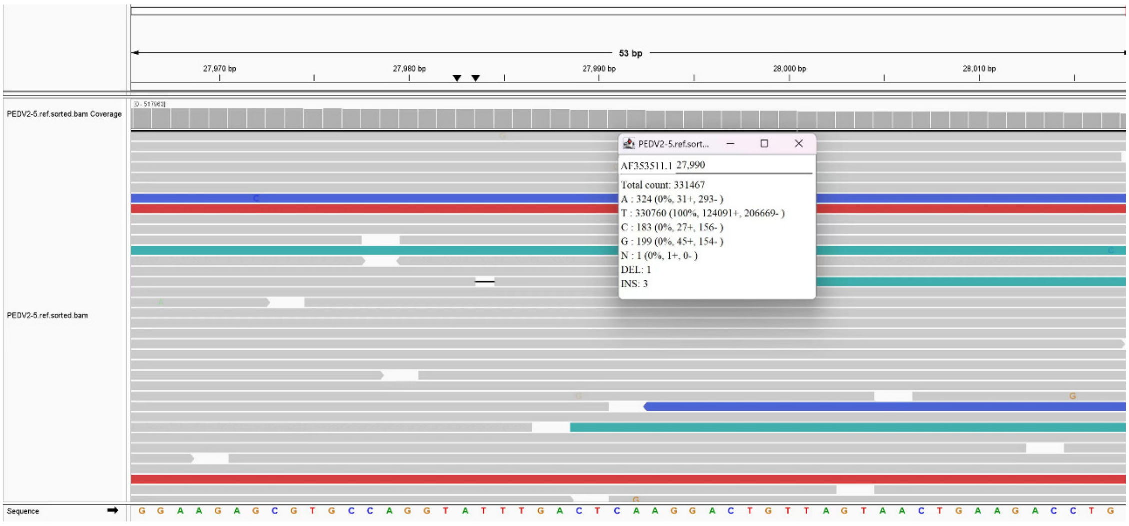

C

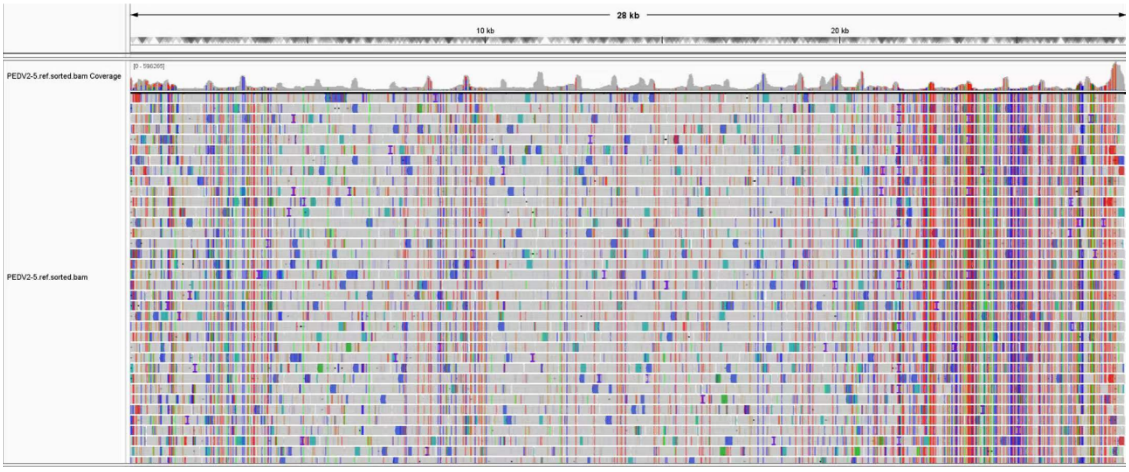

Figure S1 Read-level mapping across the recombination breakpoints of PEDV2-5 visualized by Integrative Genomics Viewer (IGV). (A) Whole-genome view showing uniform and high-depth sequencing coverage across the ~28 kb PEDV2-5 genome. (B) Local amplification at the first recombination breakpoint (~20,060 bp). The popup window indicates an ultra-high read depth (Total count: 115,205) with 100% consensus support (T: 100%), alongside specific deletion (DEL: 70) and insertion (INS: 4) events. (C) Local amplification at the second recombination breakpoint (~27,990 bp). The popup window shows a total read count of 331,467 with 100% consensus support (T: 100%), and recorded deletion (DEL: 1) and insertion (INS: 3) events. The PEDV2-5 genome was sequenced on the MGISEQ-2000 platform (PE150), achieving 100% genome coverage with an average sequencing depth of 112,153x.

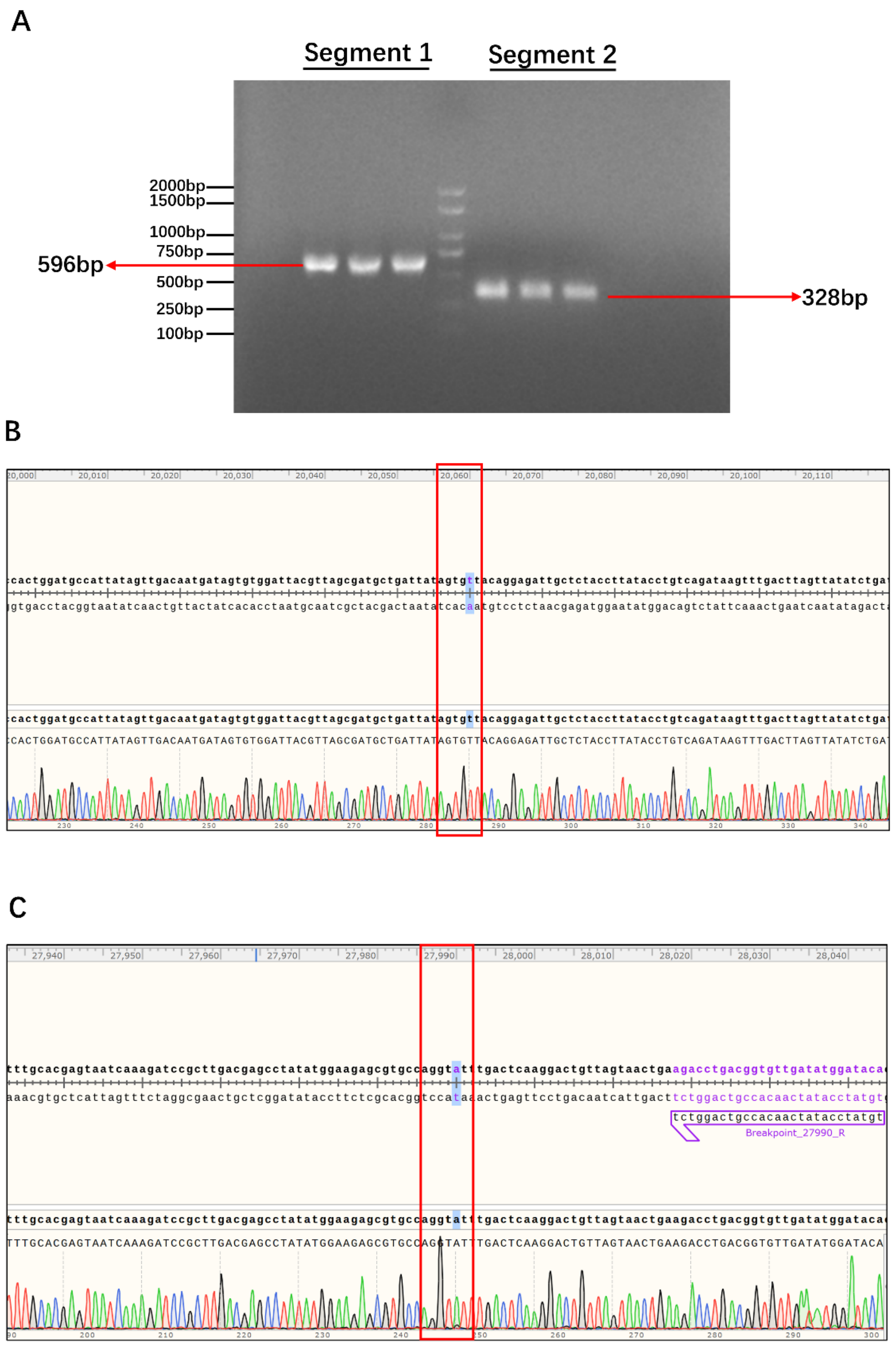

Figure S2. Figure S2 RT-PCR and Sanger confirmation for recombination breakpoint of PEDV2-5. (A) RT-PCR of segment 1 and segment 2 (contain two breakpoints respectively). (B-C) Sanger sequencing result of segment 1 (breakpoint 1, 20060 bp) and segment 2 (breakpoint 2, 27990 bp) (The red box indicates the sequencing result of the breakpoint in this segment).
